# Evaluation of an ambulatory geriatric rehabilitation program - results of a matched cohort study based on claims data

**DOI:** 10.1186/s12877-020-1415-5

**Published:** 2020-01-29

**Authors:** Simone Kiel, Carolin Zimak, Jean-François Chenot, Carsten Oliver Schmidt

**Affiliations:** 1 Department of General Practice, Institute for Community Medicine, University Medicine Greifswald, KdöR, Walther-Rathenau-Str. 48, 17475 Greifswald, Germany; 2 Department of SHIP-KEF, Institute for Community Medicine, University Medicine Greifswald, Greifswald, Germany

**Keywords:** Ambulatory geriatric rehabilitation, Geriatric multimorbidity, Health claims data, Matched cohort study, Propensity score matching

## Abstract

**Background:**

Ambulatory geriatric rehabilitation (AGR) is a multidisciplinary outpatient prevention program designed to decrease hospitalisation and dependence on nursing care in multimorbid patients ≥70 years of age. We evaluated the effectiveness of AGR compared to usual care on progression of nursing care levels, nursing home admissions, hospital admissions, incident fractures, mortality rate and total cost of care during a one-year follow-up period.

**Methods:**

Analyses were based on claims data from the health insurance company AOK Nordost. Propensity Score matching was used to match 4 controls to each person receiving the AGR intervention.

**Results:**

A total of 632 AGR participants and 2528 matched controls were included. The standardized mean difference of matching variables between cases and controls was small (mean: + 1.4%; range: − 4.4/3.9%). In AGR patients, the progression of nursing care levels (+ 2.2%, 95%CI: − 0.9 /5.3), nursing home admissions (+ 1.7%, 95%CI: − 0.1/3.5), hospital admissions (+ 1.1%, 95%CI: − 3.2/5.4), incident fractures (+ 11.1%, 95%CI: 7.3/15) and mortality rate (+ 1.2%, *p* = 0.20) showed a less favourable course compared to controls. The average total cost per AGR participant was lower than in the control group (− 353€, 95%CI: − 989€/282€), not including costs for AGR.

**Conclusions:**

Analysis based on claims data showed no clinical benefit from AGR intervention regarding the investigated outcomes. The slightly worse outcomes may reflect limitations in matching based on claims data, which may have insufficiently reflected morbidity and psychosocial factors. It is possible that the intervention group had poorer health status at baseline compared to the control group.

**Trial registration:**

German Clinical Trials Register DRKS00008926, registered 29.07.2015.

## Background

Elderly frail patients have an increased risk for hospitalisation and dependence on nursing care. To identify elderly patients in need of care, a basic geriatric assessment in ambulatory primary care was introduced in Germany in 2005 [1]. However, there are currently no widely available comprehensive outpatient rehabilitative services for geriatric patients in Germany. Inpatient geriatric rehabilitation is available but mainly used after hospitalisation, e.g. hip fractures. A systematic review showed that complex interventions can reduce the need of care in elderly [2]. Pilot programs for preventive ambulatory geriatric rehabilitation (AGR [Ambulante Geriatrische Komplexbehandlung]) were introduced within the legal frame (§ 140 Book V of the social code) in Baden-Württemberg in 1996 [3] and in Mecklenburg-Vorpommern in 2008 as well as in some other states of Germany. AGR is a multidisciplinary outpatient prevention program designed to decrease hospitalisation and dependence on nursing care in multimorbid patients aged ≥70 years. The intervention program has a duration of 4 weeks and consists of physiotherapy, ergo therapy, speech therapy, occupational therapy, social support by qualified social workers and counselling regarding aids and care. The intervention model follows the principle “outpatient before inpatient” and “rehabilitation before care”. AGR aims to strengthen and stabilise the physical and cognitive status of frail geriatric patients, enabling them to maintain independent living and thus to avoid or delay hospitalisation and dependence on nursing care. An alternative to AGR is a mobile rehabilitation program offered to patients in their homes, but this is also only available on a regional basis and has not been evaluated by a controlled study design [4]. So far, AGR has only been evaluated by uncontrolled observational studies [5, 6]. Because randomised controlled trials evaluating geriatric rehabilitation in the outpatient setting are logistically and ethically difficult to realize, we conducted a matched cohort study based on claims data.

The aim of our study was to evaluate the effectiveness of AGR with regard to progression to higher nursing care levels, nursing home admission, hospital admissions, incident fractures, mortality and health care costs within four billing periods after the intervention.

## Methods

### Study design

The study was based on claims data provided by the statutory health insurance company AOK Nordost. The intervention group consisted of AGR participants in Mecklenburg-Vorpommern during the years 2009–2013. Claims data were available on a quarterly basis, with each quarter corresponding a 3-month billing period. The observation period consisted of the four billing periods (12 months total) prior to AGR intervention. The billing period during which the 4-week AGR intervention took place represented the index period (3 months total). The four billing periods following the index period comprised the post-intervention observation period. Propensity score matching was applied to match controls and AGR participants. The study was reviewed and approved by the ethical review board of the Greifswald Medical University and the responsible authority of AOK Nordost (BB 077/14).

### Description of AGR intervention and eligibility criteria

Patients who fulfilled the eligibility criteria for AGR intervention assessed by their general practitioners were asked to participate in AGR. Eligible patients were aged 70 and older, had at least two diagnoses from a list of cardiovascular, orthopaedic, pulmonary, infectious disease, and psychiatric conditions defined by the German Geriatric Society and at least one contractually specified geriatric syndrome such as incontinence, frailty syndrome, or visual/hearing problems [7, 8]. Patients deemed suitable for AGR were referred to a special rehabilitation centre for a geriatric assessment. AGR providers then decided to include the patient in the intervention based on the geriatric assessment. The AGR intervention was tailored to the individual patient needs.

Patients were not eligible for AGR if hospital admission was indicated, AGR was deemed unreasonable, only curative rehabilitation was necessary, active participation was not possible or patient was unable to provide informed consent. The claims data did not include information about the number of patients screened for eligibility, but did not undergo AGR. .

Patients receiving AGR were treated for an average total of 20 days within 4 weeks with two to three 30-min therapy units per day. AGR was delivered in individual and group sessions with up to 15 participants. A pick up and return service for participants in rural districts was provided.

### In- and exclusion criteria from the analyses

Inclusion criteria for analysis in our study were participation in AGR during the time period 2009–2013 and available claims data. In order to evaluate treatment effects for typical AGR patients we excluded participants with rare health conditions or extremely high health care costs (*n* = 28, Table 1). Participants who died after AGR were not excluded from statistical analyses.

**Table 1:** Exclusion criteria and number of excluded AGR participants, before matching was applied *N* = 699

Exclusion criteria	Number of excluded AGR participants (*n* = 28)^b^
○ < 360 insured days in the four previous billing periods	0
○ nursing care level > 2	0
○ living in a nursing home	0
○ HIV/AIDS	0
○ chemotherapy	20
○ organ transplantation	0
○ dialysis	0
○ death including index period^a^	0
○ hospital costs without out of pocket spending at the last previous billing period ≥33.000 €	1
○ hospital costs without out of pocket spending during the four previous billing periods ≥44.000 €	3
○ ambulatory costs during the last previous billing period ≥2.200 €	3
○ ambulatory costs in the four previous billing periods ≥5.500 €	1
○ remedy costs without out of pocket spending during the four previous billing periods ≥2.200 €	1
○ costs of aid without out of pocket spending during the four previous billing periods ≥4.000€	1
○ drug costs without out of pocket spending during the four previous billing periods ≥11.000 €	2
○ total health care costs without of pocket spending during the four previous billing periods ≥44.000 €	3

^a^Index period = billing period in which the intervention took place

^b^multiple selections possible

### Matching of controls

Variables for matching cases and controls were selected based on their importance in predicting AGR participation as well as the outcomes of interest. These included use of nursing care, ambulatory care, hospital admission and inpatient diagnoses, drug prescriptions, prescription for remedies and assistive devices, as well as health care expenditures (Table 2). The four billing periods prior to the index period and the index period in which the intervention took place comprised the time frame for the variables used for matching. The index period was included in the matching procedure as it contained events (such as hospitalization) that could have motivated participation in AGR. However, within the index period we are unable to distinguish any events prior, during or after AGR. Therefore, to assess the stability of results, we excluded the index period from the matching. Any conclusions were unaffected by this approach.

**Table 2:** Matching criteria

Matching criterion	Pre matching	Propensity score	Main matching
Age	x	x	x
Sex	x		x
Area of residence		x	
Level of nursing care	x	x	x
Hospital admission (Yes/No)	x	x	x
Days spent in hospital		x	
Hospital costs without out of pocket spending	x	x	x
Ambulatory costs		x	x
Costs of remedy		x	x
Costs of medical aid		x	x
Drug costs without out of pocket spending	x	x	x
Total costs without out of pocket spending			x
*Main diagnosis before AGR*			
Cox- or gonarthrosis with endoprothesis	x	x	x
status post fracture and injuries	x	x	x
Other arthropaties	x	x	x
Osteoporosis	x	x	x
Spondylopathies and Discopathies, possibly with laminectomy	x	x	x
Pneumonia and other lung inflammations	x	x	x
Chronic obstructive pulmonary disease (COPD)		x	
Arterial obstructive disease with amputation or other surgery		x	x
Stroke and other cerebrovascular diseases		x	x
Coronary heart diseases with surgery		x	x
Delirium or other organic brain psychosis (1) only with a second main diagnosis; 2) does not apply if this diagnosis is an exclusion criterion)	x	x	x
Secondary Parkinson syndrome	x	x	x
Symptoms, effecting the nervous system and musculoskeletal system	x	x	x
*Charlson Comorbidity Index*		x	
Any Malignancy	x	x	x
Cerebrovascular disease		x	
Chronic pulmonary disease		x	
Congestive heart failure		x	
Metastatic solid tumour	x		x
Dementia	x	x	
Hemiplegia or paraplegia		x	
Mild liver disease		x	
Myocardial infarction		x	
Renal disease		x	
Peripheral vascular disease		x	
*Geriatric Multimorbidity*			
Immobility	x	x	x
Cognitive deficit		x	
Chronic pain		x	
Depression, Anxiety		x	
Incontinence		x	
Paraesthesia		x	
severe visual/hearing impairment		x	
*Falls and fractures*			
Injuries of the head			x
Injuries of the neck			x
Injuries of the thorax			x
Injuries of the abdomen, the lumbosacral region, the lumbar spine and the pelvis			x
Injuries of the shoulder and the upper arm			x
Injuries of the elbow and the under arm			x
Injuries of the wrist and the hand			x
Injuries of the hip and the thigh			x
Injuries of multiple body regions			x
Injuries of unspecific parts of the torso, extremity or other body regions			x x

Criteria which were taken into account for each stage of the matching process are marked with an X

#### The matching was performed in several steps

Since there was no clear index period for the controls, unlike the AGR patients, a pre-selection was completed on a quarterly basis. Controls were drawn with repetition for each billing periods. The pre-selection took into account the Pre Matching variables listed in Table 2. Controls were excluded if they had fundamentally different characteristics compared to AGR participants, for example other age range and health care costs outside the range of AGR participants. None of the AGR participants changed insurance providers during the study period. Therefore, only controls who did not leave the AOK Nordost were selected.

In a second step, propensity scores were calculated using a Probit-Regression model using all predictor variables listed in Table 2 (column Propensity Score).

In a third step, the actual matching of the controls using propensity-scores and other variables was performed (Table 2 Main Matching). In this step, four controls were assigned to one AGR participant without repetition. The quality of the matching was assessed using cumulative frequencies and standardized mean differences (SMD). SMD < 10% are considered satisfactory [9, 10].

### Outcomes and statistical analyses

The outcomes nursing home admission (Yes/No), nursing care levels (1/2/3), hospital admission (Yes/No) and mortality were available on a quarterly basis in claims data. Diagnoses were based on ICD-10 GM codes in claims data. Nursing care levels are defined by the German social code XI (SGB XI). The nursing care levels have been changed to nursing grades in January 2017 [11]. However, we used nursing care levels for this study, which applied during the observation period (Table 3) [8].

**Table 3:** Definitions of nursing care levels

Nursing care level	Requirements	
	Total daily help (including help in household)	Personal help (included in total daily help)
1	minimum 1,5 h	> 45 min
2	minimum 3 h	≥2 h, 3 times a day
3	minimum 5 h	≥4 h permanent help

**Fig. 1:**
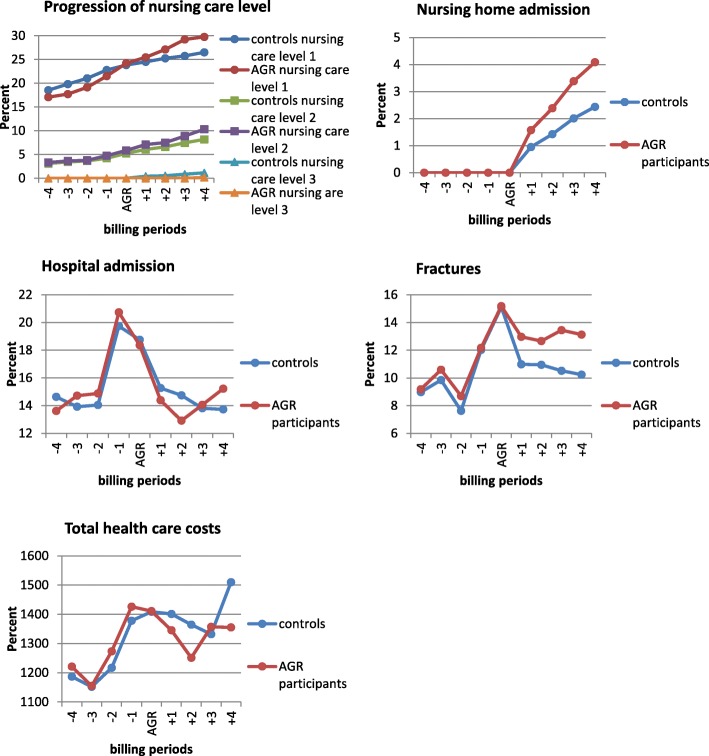
Progression of outcomes during the four previous billing periods, the index period in which the intervention took place and the four billing periods after the index period for AGR participants and controls

In addition to the diagnoses specified in Table 2, fractures (ICD S00 – T14) were also considered. The graphs (Fig. 1) represent the course of study outcomes in the intervention and control group. Effect sizes were calculated using general linear models. The predictor was the indicator variable for the AGR (yes/no). Statistical weights were calculated based on propensity scores [12] to estimate the average treatment effect. The effect sizes for the dichotomous outcomes progression to higher nursing care levels, nursing home admission, hospital admission and incident fractures were calculated using logit Link and a binomial distribution function. Differences between the groups in percent and 95% confidence limits were calculated. Changes in costs were modelled as change scores using id-Link and Gaussian distribution function. The costs for the AGR intervention were not included in the total costs. The robustness of the results was reviewed by variation of the generalized linear regression models (e.g. variation of control variables, use of different statistical weights, and exclusion of outliers in health care costs). These different approaches did not lead to a different interpretation of the results and are not reported. The analysis was performed using Stata 13.

## Results

A total of 699 geriatric patients participated in AGR during the observation period between 2009 and 2013 (mean age 79 years (SD ± 5), 72% women). Twenty-eight participants met exclusion criteria (Table 1) and 39 participants were excluded due to missing claims data. Out of a pool of 251,000 insured individuals, 2528 controls were matched to 632 AGR participants. The mean SMD of the variables after matching cases and controls was + 1.4% (range − 4.4 /3.9%). The SMD of all matching variables between AGR participants and controls can be seen in Additional file 1: Table S1. A less favourable outcome (+ 2.2%; 95% CI: − 0.9 /5.3) of progression of nursing care levels for AGR participants was observed (Table 4). In particular, a higher percentage of AGR participants entered nursing level 1 after intervention compared to non-participants (Fig. 1). More AGR participants than controls were admitted to a nursing home (+ 1.7%; 95% CI: − 0.1 / 3.5). The proportion of AGR participants with incident fractures was 11.1% (95% CI: 7.3/15) higher compared to the controls and the proportion of participants with hospital admission increased by 1.1% (95% CI: − 3.2 / 5.4). In the year after AGR, the average total cost per patient was € 353 (95% CI: € -989 / € 282) lower compared to the control group. After excluding values under the 2nd and above the 98th percentile, the difference in costs was € -144 (€ -659 / € 371). In the year after AGR intervention, 31 (4.9%) AGR participants and 93 (3.7%) controls deceased (*p*-value = 0.20).

**Table 4:** Changes of the outcomes during the one-year follow-up period

*N* = 632	% AGR	% Controls	% Difference	95% CI	p-value
progression of nursing care level	15.1	12.9	2.2	−0.9 – 5.3	0.17
nursing home admission	4.8	3.1	1.7	−0.1 – 3.5	0.06
hospital admission	39.6	38.5	1.1	−3.2 – 5.4	0.61
incident fractures	29.1	18.0	11.1	7.3–15	< 0.001
mortality	4.9	3.7	1.2	-^a^	0.20
total costs	227 €	580 €	−353 €	−989 € - 282 €	0.28
total costs without outlier	− 442 €	− 297 €	−144 €	−659 € - 371 €	0.58

^a^The difference in mortality was calculated using Chi-square test

## Discussion

### Main results

The evaluation of the AGR intervention revealed no relevant advantages compared to routine care in terms of progression of nursing care levels, nursing home admission, hospital admission, incident fractures and mortality. The course among AGR participants was slightly less favourable. The average total costs in the year after AGR were slightly lower in the intervention group but the statistical uncertainty regarding this measure was high. However, our calculation did not take into account the costs of the intervention.

### Meaning of the results and comparison with other studies

Preventing and delaying dependence on long-term care is an important public health goal given the demographic changes and the imminent shortage of nurses. Unlike a meta-analysis of 89 studies, our study did not show an advantage of multidisciplinary interventions over routine care in preventing dependence on nursing care or nursing home admission in older individuals [2]. However, the meta-analysis did not include German studies and studies were mainly related to rehabilitation after hospitalisation. The observed less favourable course of AGR participants in our analysis can be due to different reasons. One possible explanation could be that counselling regarding nursing care was offered in the intervention, which triggered an assessment for nursing care eligibility. Controls who possibly were entitled to nursing care or a higher level or nursing care might not have applied due to a lack of information and support. The available data did not comprise information on whether relatives were caring for the controls at home. This is a relevant point because 76% of people in need of care in Germany are cared for at home and of those 68% receive their care from relatives [13]. This confounder could have biased our findings.

The meta-analysis [2] showed that the number of hospital admissions could be reduced by interventions (RR 0.94; 95% CI 0.91–0.97) [2]. The results of our study show slightly more hospitalisations in the intervention group. One possible explanation is that, despite matching, there was a higher morbidity in the intervention group. In addition, we were unable to differentiate between preventable and non-preventable hospitalizations in our analyses. Conditions for which hospital admission could be prevented by an ambulatory intervention such as AGR (ambulatory care sensitive hospital admissions) include fractures, decompensated heart failure and diabetic metabolic decompensation [14]. The participant’s primary care providers retained the ability to prescribe medication during the AGR intervention. If the assigning general practitioner was not part of the AGR practice, there were limited possibilities to change prescriptions. The proportion for which this situation applies is not derivable from the available data. AGR was most likely to influence the risk of falls and fractures [15]. The proportion of AGR participants with fractures during the follow-up period of 1 year was clearly higher compared to the control group. This result is not consistent with other studies investigating patient-related clinical outcomes, which show decreased fall risk [16] and incident falls [17] as well as a significant improvement of mobility [5]. Risk factors for falls, such as deconditioning and lack of assistive devices were positively influenced by AGR. This is corroborated by a follow-up study which analysed clinical data of a subgroup of AGR participants [18]. This is another indication that the intervention group had a poorer health status than the matched peer group. Furthermore, it is necessary to continue the rehabilitation exercises in order to maintain the achieved improvements. Our data does not provide information on whether AGR participants continued exercises on their own. A long-term training program is not yet available in Germany.

A relevant influence on mortality could not be determined in the meta-analysis [2] nor in our study. However, this plays a less important role in this age group. A far more important goal is to improve and maintain quality of life. Taking into account the costs of implementing AGR, reduction in health care costs seems unlikely. From the statutory health insurance perspective, there are no relevant benefits from the implementation of the current AGR program. However, from the patient perspective there seem to be advantages [5, 6, 18]. The empirical evidence needs to be improved in order to justify the nationwide implementation of AGR.

### Strength and limitations

To the best of our knowledge, this is the first study evaluating the treatment effects of AGR in Germany using a controlled study design and a follow-up period of 1 year. Matching provided a high level of comparability with regard to the characteristics available in the claims data [9, 10]. Since neither clinical data nor psychosocial characteristics of cases and controls were available for matching, it is possible that the groups are not completely comparable. It cannot be concluded that the AGR induced more incident fractures and increased nursing home admissions. The assigning general practitioners might have used patients characteristics for AGR inclusion, unavailable through claims data. The less favourable course of AGR participants in terms of nursing care level and nursing home admission suggests that AGR participants had a poorer health status and were more vulnerable, despite adequate matching using claims data. It is possible that AGR participants, their relatives or their primary care doctor had a better knowledge of social rights, such as entitlement to higher nursing care levels, compared to controls. This study does not prove the ineffectiveness of AGR, especially since data based on a subgroup of our AGR intervention group showed consistent improvements in clinical parameters such as the Timed Up & Go Test and Barthel Index [18]. Given that a previous study based on clinical data showed improvements in a subgroup of the intervention group, claims data may not be an appropriate source of data to evaluate the efficacy of AGR. It is also possible that the clinical course of the intervention group would have been even less favourable without the AGR intervention. However, AGR is a short intervention (4 weeks) with no continuous support for patients to maintain the treatment effects. The subgroup evaluation based on clinical data showed reductions of treatment effects after 6 months follow-up, indicating the importance of implementing programs, which maintain the treatment effects [18]. The duration of the AGR corresponds to the duration of in-patient rehabilitation and the mobile geriatric rehabilitation in Germany [19, 20].

In addition, studies with more clinical and social data, which is unavailable in claims data, are needed to measure the efficacy and benefit of AGR.

## Conclusions

Analysis based on claims data showed no benefit from AGR compared to routine care in terms of reducing progression of nursing care levels, less nursing home admission, hospital admission, incident fractures and mortality. The slightly less favourable outcomes despite AGR may be due to a lack of information on morbidity and relevant psychosocial factors in claims data. A decrease in health care costs seems unlikely when considering the costs of AGR. An evaluation of AGR with a randomized controlled clinical trial would be necessary to further inform decision-maker about AGR for nationwide implementation.

## Supplementary information

**Additional file 1: Table S1.** Standardized mean differences and bias of matching variables between AGR participants and controls.

## Data Availability

Data used for analysis are anonymized claims data, which cannot be made available publicly due to regulations of the German Social Security Code. Data analysis was allowed according to § 284 Abs. 1 and Abs. 2 SGB V, and § 71 SGB V (SGB = German Social Code). According to § 67b SGB X the use of claims data and social data cannot be made available for external research unless the subjects have provided informed consent that their data may be used by someone else than their health insurance.

## Abbreviations

AGR: Ambulatory geriatric rehabilitation
CI: Confidence Interval
ICD-10 GM: International Classification of Diseases, 10. Revision, German Modification
SD: standard deviation
SMD: standardized mean differences
