# Supplementary material for: Evaluation of an ambulatory geriatric rehabilitation program - results of a matched cohort study based on claims data
Source: BMC Geriatr. 2020 Jan 29;20:30. doi: 10.1186/s12877-020-1415-5 (PMC6990495; doi:10.1186/s12877-020-1415-5)
Supplement: Supplementary file 1 — Additional file 1: Table S1. Standardized mean differences and bias of matching variables between AGR participants and controls. [file 12877_2020_1415_MOESM1_ESM.docx]

Table S1. Standardized mean differences and bias of matching variables between AGR participants and controls

| Matching variables | SMD^1^  AGR participants | SMD^1^  controls | % Bias |
| --- | --- | --- | --- |
| Sex (%) | 72.8 | 73.5 | -1.6 |
| Age (years) | 79.3 | 79.5 | -2.6 |
| Area of residence (%) | 39.7 | 39.0 | 1.4 |
| Level of nursing care at index period (%) | 35.9 | 34.4 | 2.6 |
| Level of nursing care at the third billing period prior to index period (%) | 21.7 | 22.9 | -2.9 |
| Level of nursing care at the second billing period prior to index period (%) | 4.9 | 4.2 | 3.2 |
| Hospital admission at the third billing period prior to index period (Yes/No) (%) | 14.7 | 13.9 | 2.3 |
| Hospital admission at the second billing period prior to index period (Yes/No) (%) | 14.9 | 14.0 | 2.4 |
| Hospital admission at the billing period prior to index period (Yes/No) (%) | 20.7 | 19.7 | 2.5 |
| Hospital admission at index period (Yes/No) (%) | 18.4 | 18.8 | -1.0 |
| Days spent in hospital at the third billing period prior to index period | 1.4 | 1.5 | -1.0 |
| Days spent in hospital at the second billing period prior to index period | 1.5 | 1.4 | 1.3 |
| Days spent in hospital at the billing period prior to index period | 1.9 | 1.9 | 0.1 |
| Days spent in hospital at the index period | 1.6 | 1.6 | -0.4 |
| Sum of days spent in hospital during the 4 previous billing periods | 6.2 | 6.1 | 0.9 |
| Hospital costs without out of pocket spending at the third billing period prior to index period (€) | 542 | 555 | -0.7 |
| Hospital costs without out of pocket spending at the second billing period prior to index period (€) (€) | 626 | 584 | 2.1 |
| Hospital costs without out of pocket spending at the billing period prior to index period (€) | 749 | 718 | 1.8 |
| Hospital costs without out of pocket spending at the index period (€) | 688 | 706 | -0.9 |
| Sum of hospital costs without out of pocket spending during the four previous billing periods (€) | 2515 | 2434 | 1.8 |
| Ambulatory costs at the second billing period prior to index period (€) | 206 | 200 | 3.7 |
| Ambulatory costs at the billing period prior to index period (€) | 228 | 223 | 2.9 |
| Ambulatory costs at the index period (€) | 245 | 240 | 3.5 |
| Sum of ambulatory costs during the four previous billing periods (€) | 822 | 811 | 2.4 |
| Sum of remedy costs during the four previous billing periods (€) | 55 | 49 | 4.7 |
| Sum of costs of medical aid during the four previous billing periods (€) | 173 | 166 | 2.3 |
| Sum of drug costs without out of pocket spending during the four previous billing periods (€) | 1511 | 1473 | 2.9 |
| Total costs without out of pocket spending at the third billing period prior to index period (€) | 1177 | 1175 | 0.1 |
| Total costs without out of pocket spending at the second billing period prior to index period (€) | 1298 | 1240 | 2.7 |
| Total costs without out of pocket spending at the billing period prior to index period (€) | 1458 | 1405 | 2.7 |
| Total costs without out of pocket spending at the index period (€) | 1436 | 1435 | 0.1 |
| Sum of total costs without out of pocket spending three billing periods prior to index period (€) | 5176 | 5031 | 2.8 |
| Sum of Cox- or gonarthrosis with endoprothesis during the four previous billing periods (%) | 52.1 | 53.0 | -2.0 |
| status post fracture and injuries at the third billing period prior to index period (%) | 20.4 | 20.7 | -0.9 |
| status post fracture and injuries at the second billing period prior to index period (%) | 21.9 | 21.6 | 0.9 |
| status post fracture and injuries at the billing period prior to index period (%) | 25.8 | 26.1 | -0.7 |
| status post fracture and injuries at the index period (%) (%) | 26.9 | 27.5 | -1.4 |
| Sum of status post fracture and injuries during the four billing period prior to index period (%) | 43.0 | 43.7 | -1.4 |
| Sum of other arthopathies during the four billing period prior to index period (%) | 69.1 | 70.4 | -2.8 |
| Sum of Osteoporosis during the four billing period prior to index period (%) | 35.1 | 36.0 | -1.9 |
| Sum of Spondylopathies and Discopathies. possibly with laminectomy during the four billing period prior to index period (%) | 39.1 | 38.6 | 1.0 |
| Sum of Pneumonia and other lung inflammations during the four billing period prior to index period (%) | 9.3 | 9.3 | 0.1 |
| Sum of Chronic obstructive pulmonary disease (COPD) during the four billing period prior to index period (%) | 1.7 | 1.8 | -0.9 |
| Sum of Arterial obstructive disease with amputation or other surgery during the four billing period prior to index period (%) | 18.8 | 19.6 | -1.9 |
| Sum of Stroke and other cerebrovascular diseases during the four billing period prior to index period (%) | 33.4 | 32.9 | 1.0 |
| Sum of Coronary heart diseases with surgery during the four billing period prior to index period (%) | 51.1 | 51.1 | 0.1 |
| Sum of Delirium or other organic brain psychosis during the four billing period prior to index period (%) | 23.9 | 24.5 | -1.6 |
| Sum of Secondary Parkinson syndrome during the four billing period prior to index period (%) | 3.8 | 3.2 | 3.0 |
| Sum of Symptoms. effecting the nervous system and musculoskeletal system during the four billing period prior to index period (%) | 27.4 | 25.2 | 4.9 |
| Charlson Comorbidity Index during the four billing period prior to index period (%) | 3.5 | 3.5 | -0.1 |
| Sum of any Malignancy during the four billing period prior to index period (%) | 6.2 | 6.4 | -1.0 |
| Sum of cerebrovascular disease during the four billing period prior to index period (%) | 35.3 | 34.8 | 0.9 |
| Sum of chronic pulmonary disease during the four billing period prior to index period (%) | 21.7 | 21.5 | 0.5 |
| Sum of congestive heart failure during the four billing period prior to index period (%) | 32.1 | 32.9 | -1.9 |
| Sum of Metastatic solid tumour during the four billing period prior to index period (%) | 2.4 | 2.0 | 2.4 |
| Sum of Dementia during the four billing period prior to index period (%) | 24.4 | 25.1 | -1.7 |
| Sum of Hemiplegia or paraplegia during the four billing period prior to index period (%) | 5.7 | 5.7 | 0.0 |
| Sum of Mild liver disease during the four billing period prior to index period (%) | 9.5 | 10.2 | -2.4 |
| Sum of Myocardial infarction during the four billing period prior to index period (%) | 9.3 | 9.1 | 0.8 |
| Sum of Renal disease during the four billing period prior to index period (%) | 35.6 | 34.8 | 1.6 |
| Sum of Peripheral vascular disease during the four billing period prior to index period (%) | 27.7 | 28.4 | -1.6 |
| Sum of Immobility during the four billing period prior to index period (%) | 1.0 | 0.8 | 2.2 |
| Sum of Cognitive deficit during the four billing period prior to index period (%) | 14.6 | 14.0 | 1.6 |
| Sum of chronic pain during the four billing period prior to index period (%) | 47.3 | 48.1 | -1.7 |
| Sum of Depression. Anxiety during the four billing period prior to index period (%) | 10.7 | 10.4 | 1.2 |
| Sum of Incontinence during the four billing period prior to index period (%) | 27.8 | 27.8 | 0.1 |
| Sum of Paraesthesia during the four billing period prior to index period (%) | 33.2 | 33.7 | -1.1 |
| Sum of severe visual/hearing impairment during the four billing period prior to index period (%) | 46.2 | 44.3 | 3.9 |
| Falls and fractures at fourth billing period prior to index period (%)^2^ | 9.2 | 8.9 | 0.7 |
| Falls and fractures at third billing period prior to index period (%)^2^ | 10.6 | 9.8 | 2.5 |
| Falls and fractures at second billing period prior to index period (%)^2^ | 8.7 | 7.6 | 3.9 |
| Falls and fractures at first billing period prior to index period (%)^2^ | 12.2 | 12.0 | 0.5 |
| Falls and fractures at index period (%)^2^ | 15.2 | 15.1 | 0.2 |

^1^ SMD = Standardized Mean Difference. ^2^ Falls and fractures (table 2) were combined
